# Usefulness of fecal calprotectin for the early prediction of short-term outcomes of remission-induction treatments in ulcerative colitis in comparison with two-item patient-reported outcome

**DOI:** 10.1371/journal.pone.0185131

**Published:** 2017-09-21

**Authors:** Takahiko Toyonaga, Taku Kobayashi, Masaru Nakano, Eiko Saito, Satoko Umeda, Shinji Okabayashi, Ryo Ozaki, Toshifumi Hibi

**Affiliations:** 1 Center for Advanced IBD Research and Treatment, Kitasato University Kitasato Institute Hospital, Minato-ku, Tokyo, Japan; 2 Department of Gastroenterology, Kitasato University Kitasato Institute Hospital, Minato-ku, Tokyo, Japan; Kurume University School of Medicine, JAPAN

## Abstract

**Background:**

Fecal calprotectin (FC) is well accepted as a non-invasive biomarker which objectively reflects colonic inflammation in ulcerative colitis (UC). However, its value as a marker of response during the early phase of remission induction treatment has not been well studied. The aim of this study is to evaluate the significance of FC for predicting the short-term outcomes of remission induction treatment in patients with UC.

**Methods:**

A prospective observational study was conducted among 31 patients with active UC. FC was monitored with two-item patient-reported outcome (PRO2), partial Mayo score (PMS), and Lichtiger clinical activity index (CAI) during the first 4 weeks of remission induction treatment. Clinical response was defined as a decrease in CAI of 3 or more points below baseline. Mucosal healing (MH) was defined as Mayo endoscopic subscore 0 or 1. Within-day and within-stool variability of FC were assessed during the first week of treatment.

**Results:**

In week 4-clinical responders, PRO2, PMS, and CAI significantly decreased from day 3, however, FC did not show significant reduction until week 2. Among all markers, the decrease in PRO2 at week 4 most accurately predicted MH at week 12. Within-day variability of FC was remarkably wide even at the first week in clinical responders. Within-stool variability was extremely small.

**Conclusions:**

PRO2 predicted the short-term outcomes of remission induction treatment earlier than FC possibly because of the wide within-day variability of FC in active UC.

## Introduction

Ulcerative colitis (UC) is a chronic and relapsing-remitting disease characterized by colonic inflammation with poorly defined etiology [1]. Various drugs are helpful for the treatment of UC, however, some patients are refractory to the treatment and others relapse even after once achieving remission [2]. Therefore, monitoring disease activity is an important aspect in the management of UC for evaluating treatment efficacy and predicting future relapse. Especially, in patients with acute severe disease, precise evaluation of treatment efficacy during the early phase of remission induction treatment is imperative to avoid life-threatening condition with delayed surgery. However, judging treatment efficacy during this period is difficult and has been relied on physician’s evaluation in clinical practice because of the lack of established tools for monitoring disease activity.

Increasing reports showed the usefulness of fecal calprotectin (FC) as a biomarker for distinguishing inflammatory bowel disease (IBD) consisting of UC and Crohn’s disease from irritable bowel syndrome, evaluating treatment response, and predicting MH and relapses in patients with IBD [3–6]. Thus, FC is now expected to be a non-invasive alternative to colonoscopy in the management of UC, and often used in recent clinical trials to evaluate treatment efficacy in patients with UC [7, 8]. However, it remains uncertain whether FC is useful in detecting response during the early phase, especially during the first or second week of remission induction treatment. It should also be noted that several reports showed high within-day and within-stool variability of FC in patients with active UC [9–11], which casts doubt on the usefulness of FC to monitor disease activity and detect treatment response during remission induction treatment.

In this study, we focused on the initial response to remission induction treatment in patients with UC, and evaluated the usefulness of FC in comparison with patient-reported outcome (PRO) as a marker of response during the early phase of treatment.

## Materials and methods

### Patients

A prospective observational study was conducted from September 2014 to April 2016 among 31 patients with established diagnosis of UC. For monitoring FC during remission induction treatments, 126 stool samples were collected from 27 patients with active UC prior to, 3, 7, 14, and 28 days after remission induction treatment. Total colonoscopy was performed at 12 weeks after treatment if possible to evaluate colonic mucosal status. One of 27 patients was enrolled in the study but excluded from subsequent analyses since she needed additional medical treatment before day 28 because of inadequate response to induction treatment. For evaluating within-day variability of FC, 28 fecal samples were collected from 4 patients with active UC, three of whom were enrolled twice before and a week after remission induction treatment. Fecal samples were collected throughout the day, immediately after waking-up (Morning: M), after breakfast (B), lunch (L), and dinner (D). For evaluating within-stool variability of FC, samples were collected from 2 different areas of each feces.

### Clinical activity score

Clinical activity was evaluated by two-item PRO (PRO2) extracted from Mayo score [12], CAI, and partial Mayo score (PMS) consisting of PRO2 and physician’s global assessment (PGA) score. Since there is no ‘clinical response’ established in PRO2, it was defined as a decrease in CAI at least three points below baseline as described previously [13]. Patients with a decrease in CAI less than 3 points or those who needed additional treatment because of inadequate response to initial induction treatment were defined as non-responders.

### Endoscopic severity score

All colonoscopic examinations were performed by experienced colonoscopists, blinded to the results of FC. Endoscopic severity was evaluated by Mayo endoscopic subscore (MES), and MH was defined as MES 0 or 1 as described previously [14].

### Stool samples

Collected stool samples were stored at -20°C until FC was subsequently measured by Fluoro Enzyme Immunoassay using EliA Calprotectin 2.

### Statistical analysis

All numerical data are expressed as means with SD. Differences between paired two groups were analyzed using a Wilcoxon signed-rank test. Patient's baseline characteristics were compared using Fisher’s exact test or Mann-Whitney U test. Receiver operating characteristic (ROC) analysis was used to evaluate the relationship of MH with clinical activity indices and FC, and to determine the cut-off levels that achieved the highest summation of sensitivity and specificity. Friedman’s test with post-hoc analysis of Dunn’s multiple comparison test was used to compare FC among each sample collection time in a single day. Within-day and within-stool variability of FC were evaluated by calculating coefficient of variation (CV). Pearson’s correlation coefficient was also used for evaluating within-stool variability of FC by analyzing correlation in FC between 2 separate samples from each feces. A *p* value of less than 0.05 was considered significant. The GraphPad Prism version 6.0 software (GraphPad Software Inc., San Diego, California, USA) was used for data analysis.

### Ethical considerations

The study was conducted in accordance with the Declaration of Helsinki and Good Clinical Practice. The study protocol was approved by the institutional review board at Kitasato University Kitasato Institute Hospital (approval number: 14047). All patients provided written informed consent.

## Results

### Clinical activity scores detected treatment response earlier than FC during remission induction treatment

**Fig 1:**
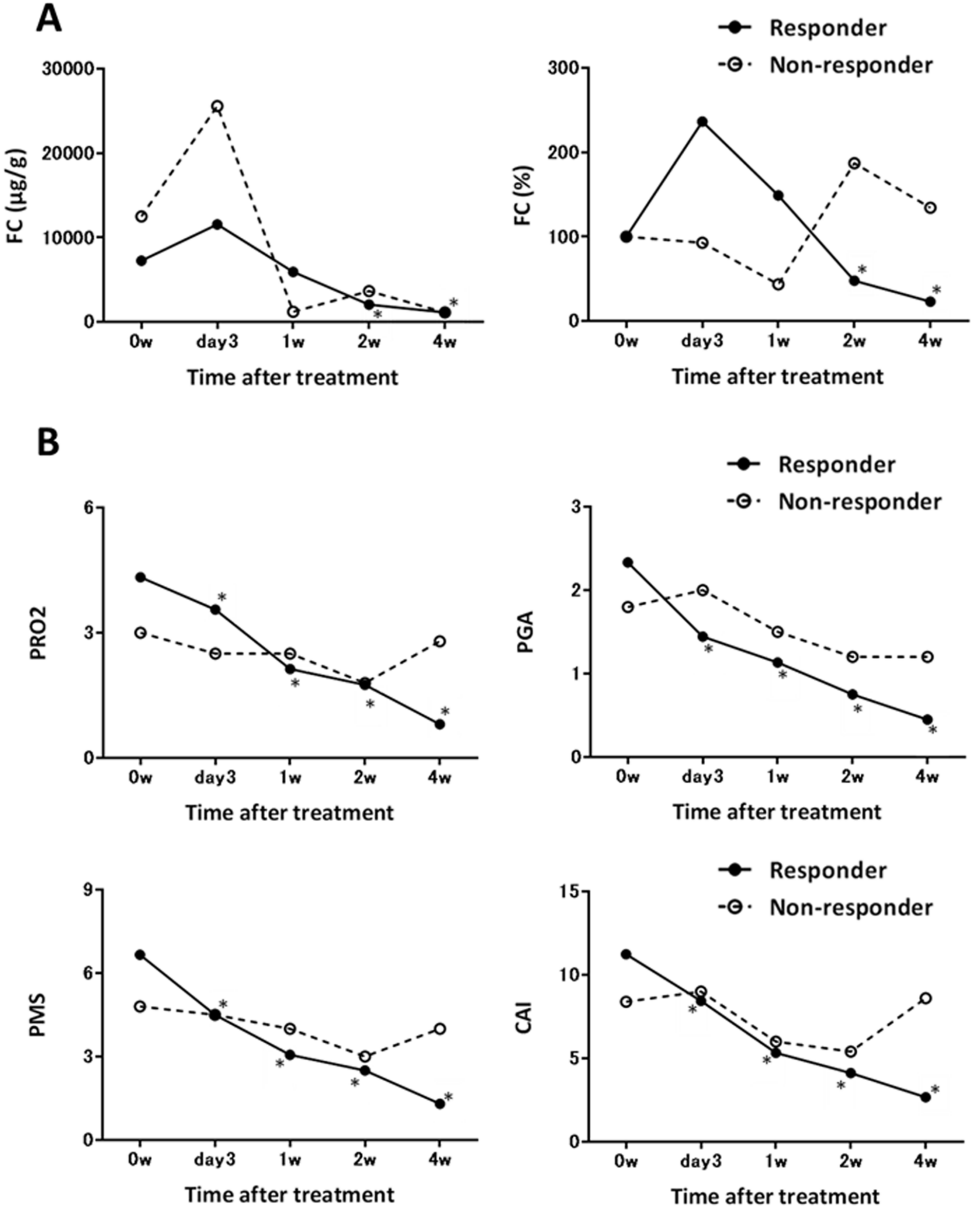
Time-dependent changes in (A) fecal calprotectin (FC), (B) two-item patient-reported outcome (PRO2) and physician’s global assessment (PGA) score from Mayo score, partial Mayo score (PMS), and Lichtiger clinical activity index (CAI) during the first 4 weeks of remission induction treatment. Each circle shows the mean value of week 4-clinical responders (open circle with solid line) and non-responders (filled circle with dotted line) at each time point. Statistical significance was determined by a Wilcoxon signed-rank test. *p<0.05 compared to the baseline of week 4-clinical responder.

Among the enrolled 27 patients, 21 patients showed clinical response to the remission induction treatment at 4 weeks after treatment. The baseline characteristics are shown in Table 1. Week 4-clinical responders showed significantly higher PMS and PRO2 at baseline than non-responders. Thirteen patients were treated with oral steroids, 3 with infliximab, 3 with adalimumab, and 2 with oral tacrolimus. These clinical responders did not show statistically significant decrease in FC until 2 weeks after treatment (FC, 236.4 ± 315.5, 149.0 ± 274.6, and 47.9 ± 67.0% of baseline at day 3, week 1, and week 2, respectively, Fig 1A). In contrast, they showed rapid and significant decrease in PRO2, PGA score, PMS, and CAI from day 3 after remission induction treatment (Fig 1B).

**Table 1:** Patient’s baseline characteristics.

	responder	non-responder	P value
total number	21	6	
Gender, male/female, n (%)	14 (66.7)/7 (33.3)	4 (66.7)/2(33.3)	1.000^a^
Age, mean (SD)	43.8 (15.8)	33.2 (11.1)	0.200^b^
Disease duration (years), mean (SD)	9.5 (8.6)	7.7 (5.2)	0.988^b^
Disease extent, left-side/extensive, n (%)	5 (23.8)/16 (76.2)	3 (50.0)/3 (50.0)	0.319^a^
Lichtiger clinical activity index, mean (SD)	11.2 (2.3)	8.8 (2.7)	0.058^b^
pMayo score, mean (SD)	6.7 (1.4)	5.0 (1.3)	0.017^b^
PRO2, mean (SD)	4.3 (1.2)	3.0 (1.0)	0.030^b^
FC (μg/g), mean (SD)	7274.0 (7888.0)	17231.3 (25239.9)	0.681^b^
Remission induction treatment (PSL/IFX/ADA/Tacrolimus)	13/3/3/2	1/3/0/3	
Concomitant treatment (5-ASA/IM/PSL/IFX)	20/9/2/1	5/4/0/0	

PRO2, two-item patient-reported outcome; FC, fecal calprotectin; PSL, prednisolone; IFX, infliximab; ADA, adalimumab; IM, immunomodulator;

^a^ Fisher’s exact test,

^b^ Mann-Whitney U test

### Decrease in PRO2 at week 4 most accurately predicted week 12-mucosal healing

**Fig 2:**
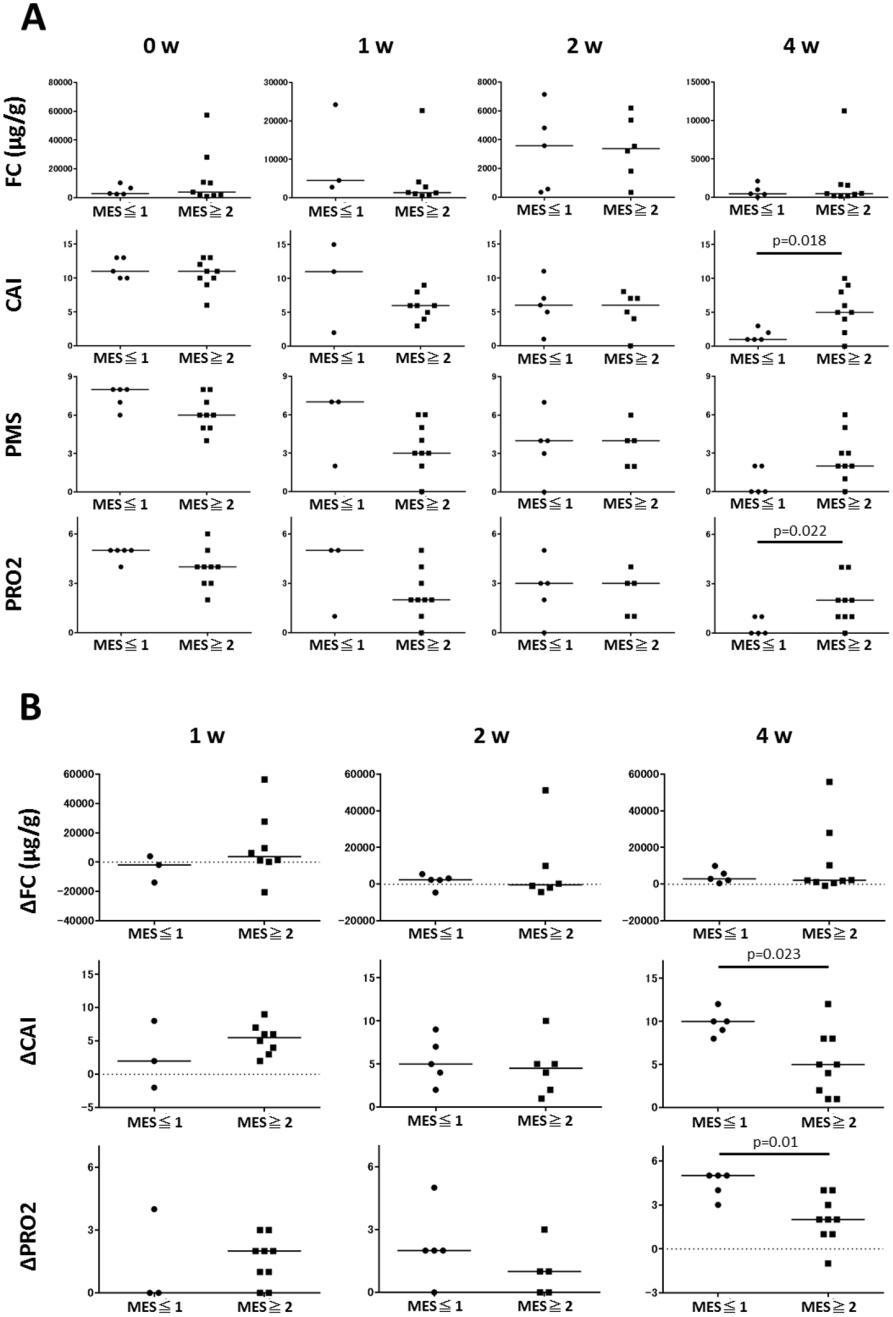
(A) Fecal calprotectin (FC) and clinical activity scores in patietns with or without mucosal healing (MH) at week 12. (B) Decrease in FC and clinical activity scores below baseline in patients with or without week 12-MH. Statistical significance was determined by a Wilcoxon signed-rank test. MES, Mayo endoscopic subscore; CAI, Lichtiger clinical activity index; PMS, partial Mayo score; PRO2, two-item patient-reported outcome; PGA, physician’s global assessment.

**Fig 3:**
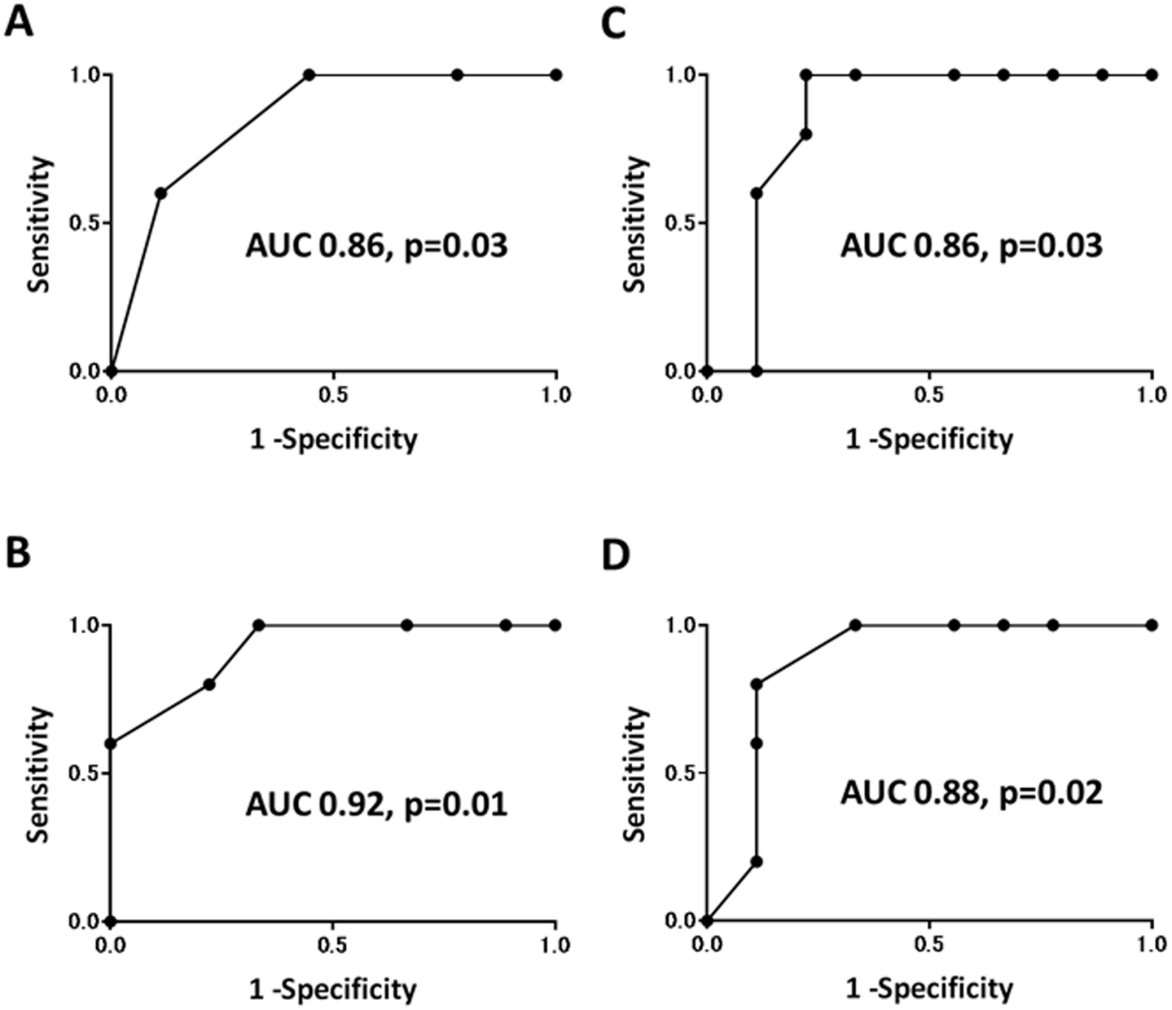
Receiver operating characteristic curves for mucosal healing versus (A) two-item patient-reported outcome (PRO2), (B) decrease in PRO2 below baseline, (C) Lichtiger clinical activity index (CAI), and (D) decrease in CAI below baseline.

Then we assessed whether the changes in FC and clinical activity scores during the first 4 weeks of remission induction treatment can predict subsequent MH. We compared FC and clinical activity scores during 4 weeks between patients with MH and those without MH at 12 weeks after treatment. As shown in Fig 2A, there was a significant difference in week 4-CAI but not PMS and FC between these two groups. When PGA item was removed from PMS, the resulting PRO2 was significantly lower at week 4 in patients with week 12-MH than those without. There was no significant difference in week 4-PGA score between two groups (0.40 ± 0.55 vs. 0.78 ± 0.67 in patients with and without week 12-MH, respectively, p = 0.41). Similarly, patients with week 12-MH showed statistically significant decrease in PRO2 and CAI but not FC below baseline at week 4 compared to those without MH (Fig 2B). ROC analysis demonstrated that week 4-PRO2 and CAI, and decrease in these scores accurately predicted week 12-MH (Fig 3A–3D). PRO2 0 or 1 predicted MH with sensitivity 1.00, specificity 0.56, positive predictive value (PPV) 0.56, and negative predictive value (NPV) 1.00. Decrease in PRO2 of 3 or more below baseline predicted MH with sensitivity 1.00, specificity 0.67, PPV 0.63, and NPV 1.00. CAI below 3 predicted MH with sensitivity 1.00, specificity 0.78, PPV 0.72, and NPV 1.00. Decrease in CAI of 8 or more below baseline predicted MH with sensitivity 0.80, specificity 0.89, PPV 0.80, NPV 0.89 (Table 2). Among these scores, decrease in PRO2 at week 4 most accurately predicted subsequent MH with the highest AUC value of 0.92 (Fig 3B).

**Table 2:** Sensitivity and specificity for week 12-mucosal healing with different cut-off of patient-reported outcome (PRO2) and Lichtiger clinical activity index (CAI) at week 4.

	Sensitivity	Specificity	Summation
**PRO2**			
< 0.5	0.60	0.89	1.49
**< 1.5**	**1.00**	**0.56**	**1.56**
< 3.0	1.00	0.22	1.22
**ΔPRO2**			
> 0.0	1.00	0.11	1.11
> 1.5	1.00	0.33	1.33
**> 2.5**	**1.00**	**0.67**	**1.67**
> 3.5	0.80	0.78	1.58
> 4.5	0.60	1.00	1.60
**CAI**			
< 1.5	0.60	0.89	1.49
< 2.5	0.80	0.78	1.58
**< 3.5**	**1.00**	**0.78**	**1.78**
< 4.5	1.00	0.67	1.67
< 5.5	1.00	0.44	1.44
**ΔCAI**			
> 4.5	1.00	0.44	1.44
> 6.5	1.00	0.67	1.67
**> 8.5**	**0.80**	**0.89**	**1.69**
> 9.5	0.60	0.89	1.49
> 11	0.20	0.89	1.09

### Fecal calprotectin showed wide within-day but small within-stool variability in active UC

**Fig 4:**
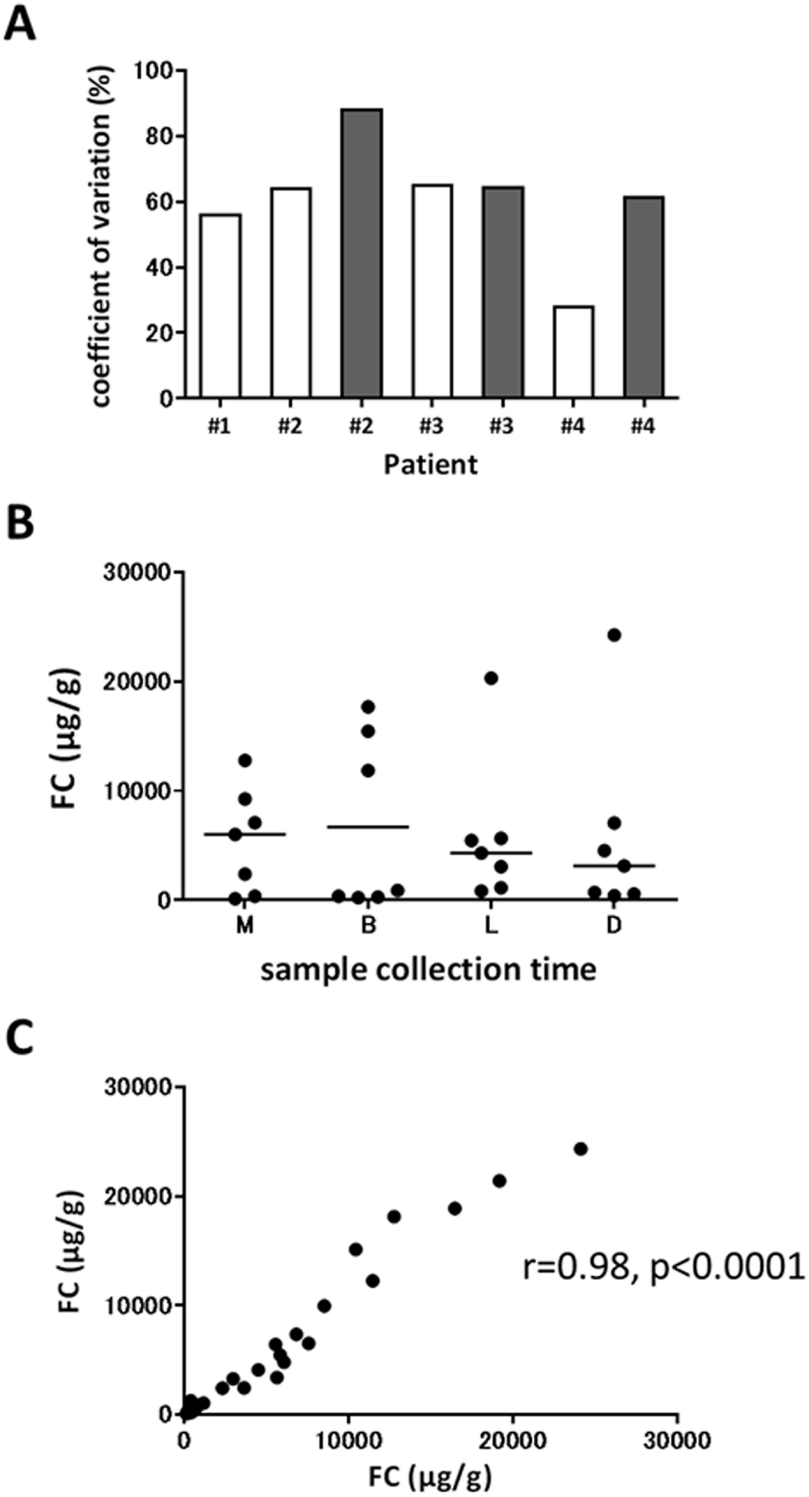
(A) Within-day variability of fecal calprotectin (FC) in 4 patients with active UC. Three of 4 patients provided fecal samples before (open box) and a week after remission induction treatment (filled box). (**B**) FC at different times in a single day. (C) Correlation between 2 samples collected from different parts of each feces. Statistical significance in FC among sample collection times was determined by Friedman’s test with post-hoc analysis of Dunn’s multiple comparison test. Pearson’s correlation coefficient was used to analyze correlation in FC between 2 separate samples from each feces. M, morning; B, breakfast; L, lunch; D, dinner.

We hypothesized that the wide intra-individual variation of FC during the early phase of induction treatment might impair its reliability for the early detection of treatment response and prediction of subsequent MH. Therefore, we investigated within-day variation by repeatedly measuring FC from same-day fecal samples submitted by patients with active UC. As shown in Fig 4A, we found wide daily variation of FC in all fecal samples with mean CV of 60.9 ± 17.7%. There was no significant correlation between FC and the sample collection times (M, 5434.0 ± 4742.3; B, 6682.7 ± 7968.0; L, 5823.7 ± 6669.3; D, 5810.6 ± 8496.3 μg/g, Fig 4B). Two of 3 patients were week 4-clinical responders, however, fecal samples provided at week 1 still presented wide within-day variability (#2, #3, and #4 in Fig 4A). To confirm that this daily variation of FC was not caused by technical issue or heterogeneity of each feces, we next assessed within-stool variability of FC by collecting 2 samples from different areas of each feces. As a result, we found small within-stool variability with mean CV of 17.6 ± 17.2%, and Pearson’s correlation coefficient showed an extremely close correlation between 2 samples from different parts of each feces (r = 0.98, p<0.0001) (Fig 4C). These results suggested that intra-individual variability might contribute to the wide variation of FC in patients with active UC during the early phase, especially within the first week, of remission induction treatment.

## Discussion

FC is an easily accessible and promising biomarker in the management of UC which might be useful in various clinical situations. However, the findings of the present study proposed the limitation of FC for predicting the short-term outcomes of induction treatment in patients with active UC. FC showed remarkably wide within-day variability in patients with active UC, and of note, it varied widely during the early phase of remission induction treatment even in clinical responders. PRO2 was superior to FC in detecting treatment response during the first week and predicting subsequent MH, which in other words, highlights the importance of close follow-up of clinical symptoms rather than relying too much on FC in this setting.

FC is currently widely accepted as a non-invasive alternative to colonoscopy which reflects colonic inflammation. FC can reflect not only the degree but also the extent of colonic inflammation, and thus is a useful tool to evaluate the status of the entire colon [15]. Furthermore, FC is reported to be useful to detect MH, although there is no consensus on the cut-off value [15, 16]. These data clearly demonstrated that FC is helpful in assessing inflammatory status in patients with UC. However, it still remains uncertain whether FC is reliable in monitoring short-term changes of disease activity especially after therapeutic intervention. Recent clinical trials often utilize FC to evaluate treatment efficacy and showed its significant decrease in clinical responders [7, 8], however, these trials compared FC prior to and several weeks after treatment. Precisely determining treatment response at the earlier time points, such as 1 or 2 weeks after treatment, is extremely important in patients with acute severe disease [17]. Nevertheless, its value as a marker of response during this period has not been well-studied yet. Theede et al. monitored FC in 16 patients with UC during 4 weeks after treatment with PSL [18]. Although they reported that all patients showed decreased FC with clinical response in the first 4 days after treatment, their data notably showed a wide variation of FC especially within 2 weeks after treatment. Similarly, Turner D. et al. measured FC in 101 pediatric patients with UC at 3 days after intravenous steroid treatment and reported that FC was not responsive to the changes in clinical activity score [19]. De Vos M et al. monitored FC every week after remission induction treatment with IFX in 53 patients with UC, and showed significant decrease in FC at week 2 but not week 1 in patients who achieved endoscopic remission at 10 weeks after treatment [20]. Consistent with these previous data, our study revealed that FC is not sensitive enough to detect response to treatment during the first week after remission induction therapy.

Then what should we rely on during this phase? Our data showed that conventional clinical activity scores, such as CAI and PMS, were more useful than FC to detect treatment response during this period. The United States Food and Drug Administration is now focusing more on PRO than these clinical activity scores as a measure of disease activity like other fields of disease [21]. Although no validated PRO exists for UC to date, Jairath V et al. extracted two-items of PRO from Mayo score, and demonstrated the usefulness of this PRO2 for evaluating treatment efficacy in patients with UC using previous clinical trial data [12]. In our study, PRO2 was superior to FC in the early detection of treatment response as with other clinical activity scores. PGA score also decreased earlier than FC in clinical responders, however, it should be noted that PGA is not specific for the disease process and might be highly susceptible to bias [12]. Because PMS was still sensitive enough for the early detection of treatment response even when PGA item was removed, PRO2 might be a more reliable measure of response than PMS in this clinical setting.

Next, we showed the usefulness of PRO2 but not FC in the early phase of induction treatment for predicting subsequent MH. Notably, we found the association of MH with early-phase CAI but not PMS and PGA score. It is possible that the higher percentage of PGA score in PMS (3 of 9 points) than CAI (5 of 21 points) impaired the power of PMS to predict future MH, which further emphasizes the necessity of eliminating PGA item from PMS. In contrast to our result, De Vos M et al. showed significant decrease in FC at 2 weeks after starting IFX in patients with UC who achieved week 10-MH compared to those who did not achieved MH [20]. These variable results might be due to the different treatment backgrounds, however, extremely wide variability of FC during the early phase of treatment in both studies imply that FC in this early period might not be suitable for predicting future MH on a case-by-case basis.

We then demonstrated wide within-day variability of FC in patients with active UC. Lasson A et al. similarly reported wide daily variability of FC with a median CV of 52% in patients with active UC. They also showed a correlation between FC and the time between bowel movement, and recommended to collect stool samples from the first bowel movement in the morning [9]. In contrast, we found no correlation between FC and sample collection times in a day. In support of our result, Calafat et al. also reported that stool sample collection from the first bowel movement in the morning did not ensure the highest or lowest within-day FC value in patients with active UC [10]. Because this wide daily variability of FC might contribute to its wide variation during early phase of induction treatment in patients with UC, FC in this period is not reliable as a marker of disease activity regardless of the sample collection time.

Limitations of this study are its small sample size and diverse induction therapies. Therefore, our results should be confirmed in a larger number of patients with a uniform treatment in the future.

In summary, our study proposed the limitation of FC as a marker of response to remission induction treatment during the early phase of treatment possibly because of its wide daily variability in patients with active UC. PRO2 was more reliable than FC in the early detection of treatment response and the prediction of future MH. These results highlight the importance of clinical symptoms rather than FC during the early phase of remission induction treatment. Our results will be helpful for the suitable utilization of FC not only in real clinical practice but also in the future clinical trials.
